# AMPK: keeping the (power)house in order?

**DOI:** 10.1042/NS20160020

**Published:** 2017-03-24

**Authors:** Claire Thornton

**Affiliations:** Perinatal Brain Injury Group, Centre for the Developing Brain, Division of Imaging Sciences and Biomedical Engineering, King's College London, St. Thomas’ Hospital, London SE1 7EH, U.K.

**Keywords:** AMP-activated protein kinase, fission, mitochondria, mitophagy, neuron

## Abstract

Metabolically energetic organs, such as the brain, require a reliable source of ATP, the majority of which is provided by oxidative phosphorylation in the mitochondrial matrix. Maintaining mitochondrial integrity is therefore of paramount importance in highly specialized cells such as neurons. Beyond acting as cellular ‘power stations’ and initiators of apoptosis, neuronal mitochondria are highly mobile, transported to pre- and post-synaptic sites for rapid, localized ATP production, serve to buffer physiological and pathological calcium and contribute to dendritic arborization. Given such roles, it is perhaps unsurprising that recent studies implicate AMP-activated protein kinase (AMPK), a cellular energy-sensitive metabolic regulator, in triggering mitochondrial fission, potentially balancing mitochondrial dynamics, biogenesis and mitophagy.

Although the brain constitutes approximately only 2% of our total body weight, it accounts for the usage of in excess of 20% of our oxygen intake and is one of the most metabolically active tissues in our bodies. Approximately 90% ATP production occurs through oxidative phosphorylation in mitochondria, therefore, for tissues with a high metabolic rate such as the brain, regulating mitochondrial health is key to sustaining cellular function [1].

Mitochondria are dynamic, double-membraned organelles that continually undergo fission, fusion and quality control (mitophagy) [2]. Long-term failure of either fission or fusion can lead to deleterious consequences and thus, a balance of these processes together with mitophagy is critical to maintaining cellular homoeostasis. Over the last 20 years, there has been a flurry of interest in discerning the molecular mechanisms regulating this mitochondrial life cycle, with significant success. Inner and outer mitochondrial membrane fusion are governed by optic atrophy (OPA)1 and mitofusin1/2 respectively whereas fission is mediated by the cytosolic protein, dynamin-related protein (Drp)1 (Figure 1) [3]. Drp1 is recruited to the mitochondrial outer membrane by binding to one of a number of mitochondrially located adaptors such as mitochondrial fission factor (Mff) and mitochondrial fission protein (Fis)1, mitochondrial dynamics proteins of 49 and 51 kDa (MiD49/51) [2,4]. Mitophagy plays a key role in the life cycle of the mitochondrion. Not only does it ensure that damaged mitochondria are neutralized, but physiological mitophagy can also regulate the number of mitochondria and their turnover [5]. In response to the decorating of the outer membrane of a depolarized mitochondrion with ubiquitin, an isolation membrane is recruited to extend round and engulf the mitochondrion, subsequently fusing with a lysosome to acidify and recycle the contents (Figure 1). Fission is frequently observed as a prelude to mitophagy as well as in the initiation of apoptosis [6]. Fusion generates a mitochondrial reticulum, allowing mitochondrial contents to mix, preventing the accumulation of mitochondrial DNA mutations as well as promoting enhanced ATP synthesis through maintenance of respiratory complexes [7].

**Figure 1:**
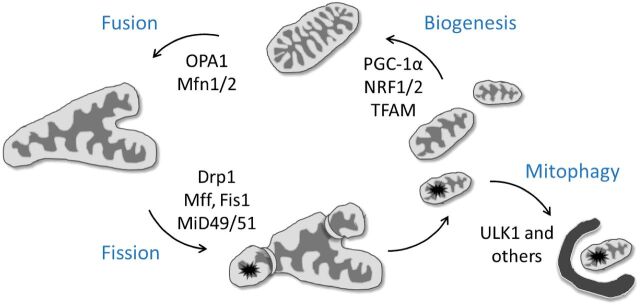
Mitochondrial dynamics Fission, fusion and mitophagy exist in a delicate balance ensuring efficient ATP production (through fusion) and degradation of damaged mitochondria (through fission and mitophagy). Up-regulation of biogenesis restores fissioned daughter mitochondria to full ATP-producing efficiency; NRF1/2, nuclear respiratory factor 1/2; TFAM, mitochondrial transcription factor A. All other abbreviations are described in the text.

The metabolic sensor, AMP-activated protein kinase (AMPK) is a serine/threonine protein kinase existing as a heterotrimer of catalytic (α1/α2) and regulatory subunits (β1/β2 and γ1/γ2/γ3). The 12 possible heterotrimers exhibit tissue and potentially functional specificity [8], and all can be activated by binding of AMP/ADP to the AMPKγ subunit and phosphorylation by one of two upstream kinases, liver kinase B (LKB)1 or calcium/calmodulin-dependent protein kinase kinase (CaMKK)β. AMPK is activated in response to depletion of ATP or alterations in intracellular calcium concentrations, and acts to shut down ATP-consuming, anabolic pathways and promoting ATP-generating, catabolic pathways [9].

As a monitor of cellular and whole body energy status [10], it is probably unsurprising that a recent elegant study in *Science* from Reuben Shaw's laboratory places AMPK at the heart of the regulation of mitochondrial dynamics. Using CRISPR modification to delete AMPKα1 and/or AMPKα2 *in vitro*, they discovered that AMPK was critical to the fission response induced by mitochondrial poisons targeting the electron transport chain, such as rotenone; an absence of AMPK complexes resulted in an absence of fission [11]. Interestingly, direct pharmacological activation of AMPK was sufficient to promote fission, and equally effective regardless of whether AMPKα1 or AMPKα2-containing complexes were expressed. Toyama et al. further identified Mff as a substrate for AMPK phosphorylation, echoing a similar finding from the Sakamoto's laboratory where Mff was isolated in a proteomics screen for AMPK substrates in activated hepatocytes [12]. Mff is located on the outer mitochondrial membrane and acts to recruit Drp1, which subsequently oligomerizes and constricts the mitochondrion, leading to fission [13]. Toyama et al. [11] showed that AMPK-mediated phosphorylation of Mff at S155 and S172 is required for Drp1 recruitment, and *in vitro* studies of primary hepatocytes and primary cortical neurons imply that this mechanism could be ubiquitous. This study, therefore, identifies AMPK as the bridge between bioenergetic crisis and the induction of mitochondrial fission.

So is the fission-inducing action of AMPK conferring advantage or disadvantage to a neuron under siege? Reducing its ATP-producing capability during injury by dividing up mitochondria may not, at first glance, seem like a beneficial strategy, especially if it leads to the induction of apoptosis. However, fissioned mitochondria have a crucial role in neurons, as discrete mitochondria are required for axonal transport via microtubules to synaptic terminals, providing a local ATP source to fuel signal progression [14]. But as mentioned earlier, fission is also required as an initiating step in the progression of mitophagy, in which damaged mitochondria are cleared away, preventing elevated production of reactive oxygen species (ROS) as well as contributing to the restoration of efficient Ca^2+^-buffering capacity.

Coincidently, among its large library of metabolic substrates, AMPK phosphorylates Unc-51-like kinase 1 (ULK)1, a regulator of autophagy [15,16],which can itself regulate AMPK activity by phosphorylation [17]. Inhibiting AMPK-mediated ULK1 phosphorylation leads to accumulation of damaged mitochondria and prevention of mitophagy [15,18]. In addition, AMPK regulates peroxisome proliferator-activated receptor (PPAR)γ co-activator (PGC)-1α, a master transcriptional activator of mitochondrial biogenesis, although whether this regulation is direct (by phosphorylation) or indirect (via SIRT1 deacetylation) is currently still debated [19,20]. Taken together, it is tempting to speculate that after metabolic insult, AMPK facilitates mitochondrial health in three distinct ways (Figure 2). Initially, in response to perturbations of the electron transport chain and subsequent ATP depletion, AMPK phosphorylates Mff, inducing fission. Concomitantly, activated AMPK can promote mitophagy of these small, damaged mitochondria through ULK1 phosphorylation as well as restore the daughter mitochondrion to full ATP production through PGC-1α-mediated mitochondrial biogenesis. The suggested benefits imply a renewal of robust mitochondrial content within the cell, limiting damage and able to withstand cellular ATP demands. Such a mechanism may be in part responsible for the role of AMPK in pre-conditioning, a paradigm in which a sub-lethal stress is evoked prior to the onset of lethal injury (Figure 2). This sub-lethal exposure renders cells more resistant to injury and pre-activation of AMPK has provided significant survival benefits in a wide range of ischaemic injuries [21–25].

**Figure 2:**
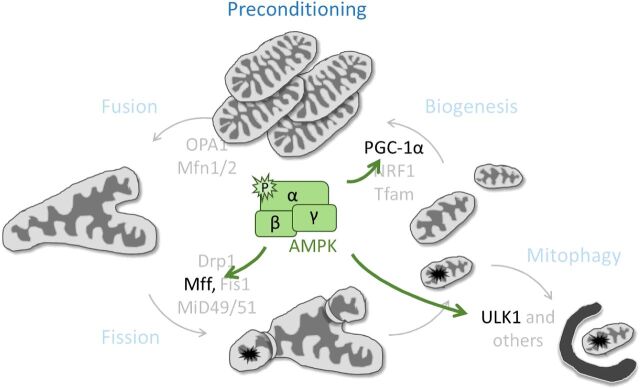
AMPK regulates mitochondrial health and may mediate pre-conditioning Superimposed on this continuous mitochondrial life cycle is activated AMPK, which phosphorylates substrates at key points potentially tailoring a robust cellular response to pathological stimulus.

However, while this wholesome scenario (in which AMPK promotes not only the sweeping away of ROS-producing mitochondria but also the energy efficiency of cell) is tempting, it is worth remembering that both fission and AMPK activation have independently been reported to mediate neurodegeneration. Fission occurs as an early event in a number of neurodegenerative diseases including Alzheimer's disease [26] and Huntington's disease [27] as well as occurring after brain trauma such as stroke or neonatal hypoxic-ischaemic injury [28,29], environments in which AMPK is known to be activated [30–32]. Indeed, inhibitors of post-injury fission, such as mDivi-1 and p110, have proved successful as neuroprotectants. As Drp1 interacts with a multitude of mitochondrial adaptors, further work is clearly required to determine differential contributions of these Drp1 binding partners, e.g. Fis1 in Huntington's disease [33]. It is also interesting to speculate that these findings might be a matter of timing. Inducing mitophagy too soon after injury could easily prove injurious if ATP production from damaged mitochondria (albeit limited) is more valuable to neuronal survival than the concomitant ROS accumulation. In such circumstances, delayed induction of mitophagy (through inhibition of fission) may well prove more and more advantageous treatment strategy. AMPK activity is also known to be up-regulated after brain injury in which there is ATP depletion. Not surprisingly then, the first evidence that AMPK activation may be deleterious was provided in a stroke model where it was found that pharmacological inhibition of AMPK or ablation of AMPKα2 (but surprisingly not AMPKα1) reduced infarct size [34,35]. Furthermore, and of specific interest here, recent studies have suggested that AMPK activation can induce fusion [36], that AMPK acts downstream of Drp1 [37] or even that AMPK directly phosphorylates Drp1 [38], inhibiting its capability for scission. These contradictory observations may very well be resolved once the contribution of the upstream kinases LKB1 and CaMKKβ, AMPK subunit specificity, physiological compared with pathological AMPK stimuli (direct or indirect) and chronic compared with acute AMPK activation are deciphered. What is becoming clear though is that AMPK activation and mitochondrial dynamics are delicately intertwined, and modulating AMPK activity to maintain mitochondrial and, by extension, neuronal health remains an intriguing therapeutic possibility.

## Abbreviations

AMPK: AMP-activated protein kinase
CaMKK: calcium/calmodulin-dependent protein kinase kinase
Drp: dynamin-related protein
Fis: mitochondrial fission protein
LKB: liver kinase B
Mff: mitochondrial fission factor
MiD49/51: mitochondrial dynamics proteins of 49 and 51 kDa
OPA: optic atrophy
PGC-1α: PPARγ co-activator-1α
PPAR: peroxisome proliferator-activated receptor
ROS: reactive oxygen species
ULK1: Unc-51-like kinase 1
